# Utility of the lateral flow urine lipoarabinomannan tuberculosis assay in patients with advanced HIV disease at antiretroviral therapy centres in Mumbai, India

**DOI:** 10.1371/journal.pone.0273970

**Published:** 2022-09-14

**Authors:** Shrikala Acharya, Prashant Deshpande, Edwin Sam Asirvatham, Amol Palkar, Charishma Jones Sarman, Chinmay Laxmeshwar, Maninder Singh Setia, Dhirubhai Rathod, Sagar Koli, Jayesh Dale, Vijay Yeldandi, Ramesh Allam, Reshu Agarwal, Sanjeev Verma, Sunita Upadhyaya, Melissa Nyendak

**Affiliations:** 1 Mumbai Districts AIDS Control Society (MDACS), Mumbai, Maharashtra, India; 2 UW-International Training and Education Center for Health, New Delhi, India; 3 Society for Health Allied Research & Education India (SHARE INDIA), Medchal-Malkajgiri District, Telangana, India; 4 PATH India, Mumbai, Maharashtra, India; 5 Consultant Dermatologist and Epidemiologist, Mumbai, Maharashtra, India; 6 Division of Global HIV and TB, Centers for Disease Control and Prevention (CDC), New Delhi, India; National Tuberculosis Institute, GUINEA

## Abstract

**Background:**

People with Advanced HIV Disease (AHD) are at higher risk of TB coinfection and mortality. However, there are challenges in TB diagnosis with the currently recommended diagnostic tools. WHO recommends lateral flow urine lipoarabinomannan (LF-LAM) assay to assist TB diagnosis among AHD patients. We assessed the utility and acceptability of using urine LF-LAM assay for TB diagnosis among patients at public Antiretroviral Therapy (ART) Centres in Mumbai.

**Methods:**

The cross-sectional study was conducted among adult AHD patients accessing care from 17 ART centres during November,2020-June, 2021. Urine LF-LAM was offered as routine care for eligible patients in combination with standard diagnostic tests. We calculated the proportion of positive LF-LAM results by CD4 categories and TB symptoms and performed multivariable logistic regression to determine the factors associated with LF-LAM positivity.

**Results:**

Among 2,390 patients, the majority (74.5%) had CD4 between 101–200 cells/mm^3^. The mean age was 43.7 years (SD:10.6), 68.6% were male, 8.4% had TB symptoms and 88.0% were on ART. The overall proportion of patients with urine LF-LAM positive results was 6.4%. Among PLHIV with CD4≤100 cells/mm^3^, the positivity was 43.0% and 7.7% in symptomatic and asymptomatic patients, respectively. Among PLHIV with a CD4>100 cells/mm^3^, the positivity was 26.7% and 2.7% in symptomatic and asymptomatic patients respectively. Urine LF-LAM positivity was higher among inpatients, ART naïve, patients on treatment for <6 months, symptomatic and in WHO clinical stage III/IV of HIV disease as compared to the reference categories. We detected an additional 131 TB cases with urine LF-LAM in combination with the standard diagnostic tests.

**Conclusion:**

The study demonstrated the utility of urine LF-LAM for TB diagnosis among AHD patients and the simple, user-friendly test was acceptable as part of routine care. Inclusion of urine LF-LAM test in the current diagnostic algorithm may facilitate early TB diagnosis among AHD patients.

## Background

Globally tuberculosis (TB) remains the leading cause of death with 10 million incident cases and 1.5 million deaths in 2020 [1, 2]. In people living with HIV (PLHIV), TB is the most common opportunistic infection (OI) responsible for hospitalization and deaths despite increased access to antiretroviral therapy (ART) [3, 4]. The risk of developing TB is 18 (15–21) times higher among PLHIV [5]. Global estimates indicate a 44% gap in case detection among people with HIV associated TB [6]. India has the second-highest burden of HIV-TB co-infected cases accounting for 9% of the global caseload with an estimated 92,000 HIV- TB co-infected cases and 9,700 deaths in 2020 [7].

WHO defines advanced HIV disease (AHD) for adults as having CD4 count <200 cells/mm^3^ and/or WHO clinical stage III or IV [8]. Diagnosis of TB in patients with AHD is often difficult due to higher rates of paucibacillary and extrapulmonary TB (EPTB) disease. The majority (60%) of EPTB among AHD patients do not have traceable amounts of TB bacteria in sputum, and between 24% and 62% of HIV-infected patients have sputum smear-negative results [9, 10].

This situation underscores the need for diagnostic tests that are able to detect disease without requiring sputum collection. WHO recommends Lateral Flow urine lipoarabinomannan (urine LF-LAM) assay, a rapid point of care test to assist in the diagnosis of TB in patients with advanced HIV-induced immunosuppression, to address the associated TB morbidity and mortality [11]. Because of the low sensitivity of sputum Xpert MTB/RIF in HIV-positive people, the inability to produce sputum and low bacillary burden in sputum, the urine LF-LAM test may improve the detection of TB cases, particularly among those missed by rapid molecular diagnostic tests [12–14].

The National AIDS Control Programme (NACP) in India has been implementing “single window services” for TB prevention, diagnosis and management for PLHIV at ART centres since 2016 [15]. The NACP guidelines recommend intensified TB case finding through screening of all PLHIV for four symptoms (4S)—current cough, fever, weight loss and/or night sweats, during every visit to ART Centres [16]. PLHIV with any of the 4 TB symptoms are referred for sputum cartridge-based nucleic acid amplification (CBNAAT) using Xpert MTB/RIF test and other radiological investigations for TB diagnosis [17].

WHO recommends urine LF-LAM to assist in the diagnosis of active TB among patients with AHD with signs and symptoms of TB among seriously ill patients, and in inpatient and outpatient settings. Additionally, WHO recommends LF-LAM irrespective of symptoms for inpatients with AHD, and among symptomatic outpatients with a CD4 count of 100–200 cells/mm^3^. It recommends against using LF-LAM in the diagnosis of active TB among patients with a CD4 count of 100–200 cells/mm^3^ without TB symptoms in outpatient settings [11]. Urine LF-LAM is being considered as a diagnostic test in combination with existing tests for the diagnosis of HIV-associated TB [18].

The 15^th^ Technical resource group (TRG) meeting in September 2020 at National AIDS Control Organization (NACO), India recommended urine LF-LAM test for all patients with AHD regardless of TB symptoms, and patient settings as a modification to the WHO 2019 recommendation, with suggestions for operational feasibility studies. The urine LF-LAM test has been included in Advanced Disease Management (ADM) package in National Operational Guidelines for ART services, 2021 [16, 19, 20]. At present, Alere® Determine TB LAM Antigen Lateral Flow Assay is not licenced for commercial use in the country and there is a paucity of data on the use of LF-LAM tests among PLHIV patients in India. In this context, we assessed the utility of the urine LF-LAM assay for the diagnosis of TB among patients with AHD and its acceptability among health care staff at public ART centres in Mumbai.

## Methods

### Study setting

The study was conducted in 17 publicly funded ART Centres in Mumbai, India, under the aegis of Mumbai Districts AIDS Control Society (MDACS) responsible for the implementation of HIV control activities. During the study period, around 38,000 PLHIV were registered for HIV treatment and care. The ART centre team comprises multi-disciplinary health staff including a qualified physician, nurse, pharmacist, counsellor, data manager and a community representative. Of the 17 ART centres selected for the study, 6 centres were located in tertiary, 8 in secondary and 3 in primary health care facilities.

All adults (≥18 years) with AHD (CD4<200 cells/mm^3^ or with WHO clinical stage III or IV) were eligible for enrolment. We included persons with AHD from inpatient and outpatient settings who were either on ART or newly presenting for treatment initiation. PLHIV on current TB treatment, or who completed TB treatment in the past three months were excluded.

### Screening and diagnosis of TB by urine LF-LAM and sputum Xpert MTB/RIF

ART centre staff, including the medical officer, nurse, counsellor and laboratory technician were trained on the usage of urine LF-LAM test among AHD patients. The nurses of the ART centres provided necessary information related to the purpose of study, testing procedures, privacy and assurance of confidentiality of details to the study participants. A written informed consent using the prescribed informed consent form was obtained by the nurses.

Fresh urine specimens were collected and processed at the ART centre by the laboratory technician as per standard operating procedures (SOP) and the manufacturer kit insert. We used the *in vitro* Alere® Determine TB LAM Antigen lateral flow assay (Abbott Laboratories, Lake Bluff, USA) to visually read qualitative immunocapture assay for the detection of lipoarabinomannan (LAM) antigen of mycobacteria in human urine. The results were interpreted by trained staff and were made available to the patients and the medical officers within two hours of sample collection. PLHIV with any of the 4 TB symptoms were referred for sputum Xpert MTB/RIF and other diagnostic tests as per routine practice.

### Data collection, data management and analysis

Routine programmatic data from the patient records at ART centres consisting of demographic details (age, gender), patient setting (outpatient and inpatient), ART status (on ART and ART naïve), presence of TB symptoms, duration of ART, CD4 count, most recent viral load count (if available), LF-LAM results, and sputum Xpert MTB/RIF results were collated. All data were entered in Microsoft Excel (Version MSO (16.0.10383.20027)) and analysed using IBM SPSS Statistics for Windows (Version 26.0) [21]. Mean, standard deviation, median and interquartile range as appropriate were calculated for continuous variables. Frequency and percentages were calculated for categorical variables.

To determine the association between TB symptoms (symptomatic and asymptomatic) and independent variables, chi-square test or Fisher’s exact test was used. Similarly, independent t-test and Mann-Whitney U test were used for continuous variables based on the distribution. Univariate binary logistic regression was used to determine the association between urine LF-LAM positivity and socio-demographic characteristics, ART status, treatment duration, viral load count, presence of TB symptoms, WHO clinical staging and CD4 count. Odds ratio and 95% confidence intervals were reported. Multivariable logistic regression was performed to assess the factors associated with urine LF-LAM positivity. A p-value of less than 0.05 was considered statistically significant.

ART centre staff including medical officers, counsellors, nurses and lab technicians from 10 ART centres were interviewed in person by the trained study team using a semi-structured interview schedule. The study team assessed the acceptability and challenges of using urine LF-LAM in the program setting. The responses were recorded in text using double quotations as per standards of presenting qualitative data.

### Ethical considerations

The study protocol was approved by the Ethics committee of Mumbai Districts AIDS Control Society (Ref: MDACS/3230/STI Dtd 28/10/2020). The study also received a non-research determination from the Scientific Integrity Branch of the Division of Global HIV and TB, CDC, Atlanta.

## Results

### Demographic, and clinical characteristics of the patients

Among the 2,602 adults with AHD eligible for participation in the study, we enrolled 2,390 (91.8%) persons for urine LF-LAM testing between November 2020 and June 2021. Of them, 201 (8.4%) were symptomatic for any of the 4 TB symptoms and 2,189 (91.6%) were asymptomatic.

The mean age of the participants was 43.7 years (SD: 10.6), 68.6% were male, the majority (50.6%) were 45 years and above. Twelve per cent were treatment naïve and among the 88.0% who were on ART, 68.2% were on ART for more than 24 months. The median duration on ART was 51.8 months (IQR: 18.6–98.7). The cohort was predominantly outpatients (98.1%; 2,344/2,390) and 24.3% (n = 580) had CD4 count ≤100 cells/mm^3^. Of the 1,682 persons on ART who underwent viral load (VL) testing, 71.5% were virally suppressed (VL <1,000 copies/ml). The majority (79.9%) were in WHO clinical stage I (Table 1).

**Table 1:** Distribution of demographic, clinical and treatment characteristics by CD4 count.

Patient Characteristics		CD4 ≤100 cells/mm^3^ n = 580		CD4>100 cells/mm^3^ n = 1,810		Total N = 2,390	
		n	%	n	%	n	%
**Age group (years)**	*18 to 24*	37	6.4	83	4.6	120	5.0
	*25 to 34*	83	14.3	237	13.1	320	13.4
	*35 to 44*	178	30.7	563	31.1	741	31.0
	*45 and above*	282	48.6	927	51.2	1209	50.6
**Gender**	*Male*	381	65.7	1259	69.6	1640	68.6
	*Female*	195	33.6	541	29.9	736	30.8
	*Transgender*	4	0.7	10	0.6	14	0.6
**Patient settings**	*Inpatient*	26	4.5	20	1.1	46	1.9
	*Outpatient*	554	95.5	1790	98.9	2344	98.1
**ART status **	*ART Naïve*	127	21.9	159	8.8	286	12.0
	*On ART*	453	78.1	1651	91.2	2104	88.0
**Duration on ART in months (n = 2,104)**	*< 6*	100	22.1	176	10.7	276	13.1
	*6 to 12*	29	6.4	110	6.7	139	6.6
	*13–24*	57	12.6	198	12.0	255	12.1
	*> 24*	267	58.9	1167	70.7	1434	68.2
**TB Symptoms (4S screening)**	*Symptomatic*	100	17.2	101	5.6	201	8.4
	*Asymptomatic*	480	82.8	1709	94.4	2189	91.6
**Viral Load count (n = 1,682)**	*< 1000*	156	53.4	1046	75.3	1202	71.5
	*≥ 1000*	136	46.6	344	24.7	480	28.5
**WHO clinical stage (n = 2,385)**	*Stage I*	422	73.0	1484	82.1	1906	79.9
	*Stage II*	89	15.4	236	13.1	325	13.6
	*Stage III*	24	4.2	35	1.9	59	2.5
	*Stage IV*	43	7.4	52	2.9	95	4.0
**Urine LF-LAM results **	*Positive*	80	13.8	73	4.0	153	6.4
	*Negative*	500	86.2	1737	96.0	2237	93.6
**Xpert MTB/RIF (n = 138) **	*Positive*	19	27.1	10	14.7	29	21.0
	*Negative*	51	72.9	58	85.3	109	79.0

We stratified the patients based on CD4 count (≤100 and > 100 cells/mm^3^) and TB symptoms (symptomatic and asymptomatic) (Table 2). Older people with CD4 ≤100 cells/mm^3^ were less likely to be symptomatic for TB (p = 0.01). The proportion of persons with TB symptoms was significantly higher among inpatients (p<0.01), ART naïve (p<0.01), PLHIV on ART for <6 months (p = 0.01; p = 0.04) and PLHIV in WHO clinical stage III and IV (p<0.01) in both the CD4 groups as compared to outpatients, PLHIV on treatment and for more than 6 months, asymptomatic and PLHIV in WHO stage III and IV respectively.

**Table 2:** Distribution of demographic, clinical and treatment characteristics.

	CD4 ≤100 (n = 580)			CD4 >100 (n = 1,810)			
Description	Symptomatic (n = 100)	Asymptomatic (n = 480)	P value	Symptomatic (n = 101)	Asymptomatic (n = 1709)	P value	All Patients (n = 2390)
	n (%)	n (%)		n (%)	n (%)		N (%)
**Mean Age in years (SD)**	40.73 (SD:11.1)	43.4 (SD:10.8)	0.02	41.8 (SD:10.1)	44.0 (SD:10.4)	0.04	43.7 (SD: 10.6)
**Age category (in years)**							
*18 to 24*	9 (24.3)	28 (75.7)	0.015	5 (6.0)	78 (94.0)	0.17	120 (5.0)
*25 to 34*	23 (27.7)	60 (72.3)		17 (7.2)	220 (92.8)		320 (13.4)
*35 to 44*	30 (16.9)	148 (83.1)		38 (6.7)	525 (93.3)		741 (31.0)
*45 and above*	38 (13.5)	244 (86.5)		41 (4.4)	886 (95.6)		1209 (50.6)
**Gender **							
*Male*	66 (17.3)	315 (82.7)	1.00	65 (5.2)	1194 (94.8)	0.37	1640 (68.6)
*Female*	34 (17.4)	161 (82.6)		36(6.7)	505 (93.3)		736 (30.8)
*Transgender*	0 (0.0)	4 (100.0)		0 (0.0)	10 (100.0)		14 (0.6)
**Patient settings**							
*Inpatients*	15 (57.7)	11 (42.3)	<0.01	13 (65.0)	7 (35.0)	<0.01	46 (1.9)
*Outpatients*	85 (15.3)	469 (84.7)		88 (4.9)	1702 (95.1)		2344 (98.1)
**ART status **							
*ART naïve*	42 (33.1)	85 (66.9)	<0.01	25 (15.7)	134 (84.3)	<0.01	286(12.0)
*On ART*	58 (12.8)	395 (87.2)		76 (4.6)	1575 (95.4)		2104(88.0)
**Duration of ART in Months (Median (IQR)**	17.9 (0.8–66.3)	40.5 (11.3–90.9)	0.01	41.7 (10.5–86.7)	56.0 (20.9–101.4)	0.01	51.8 (18.6–98.7)
**Duration of ART (months) (n = 2,104) **							
*< 6 months*	21(21.0)	79 (79.0)	0.01	15 (8.5)	161 (91.5)	0.04	276 (13.1)
*6 to 12*	2 (6.9)	27 (93.1)		7 (6.4)	103 (93.6)		139 (6.6)
*13–24*	10 (17.5)	47 (82.5)		7 (3.5)	191 (96.5)		255 (12.1)
*> 24*	25 (9.4)	242 (90.6)		47 (4.0)	1120 (96.0)		1434 (68.2)
**VL count (n = 1,682)**							
*< 1000*	6 (3.8)	150 (96.2)	0.006	38 (3.6)	1008 (96.4)	0.90	1202 (71.5)
*≥ 1000*	17 (12.5)	119 (87.5)		12 (3.5)	332 (96.5)		480 (28.5)
**WHO clinical stage at enrolment (n = 2,385)**							
*Stage I*	33 (7.8)	389 (92.2)	<0.01	41 (2.8)	1443 (97.2	<0.01	1906 (79.9)
*Stage II*	20 (22.5)	69 (77.5)		8 (3.4)	228 (96.6)		325 (13.6)
*Stage III*	12 (50.0)	12 (50.0)		16 (45.7)	19 (54.3)		59 (2.5)
*Stage IV*	34 (79.1)	9 (20.9)		36 (69.2)	16 (30.8)		95 (4.0)
**LF- LAM results (column%)**							
*Positive*	43 (43.0)	37 (7.7)	<0.01	27 (26.7)	46 (2.7)	<0.01	153 (6.4)
*Negative*	57 (57.0)	443 (92.3)		74 (73.3)	1663 (97.4)		2237 (93.6)
**Xpert MTB/RIF results (n = 138) (column%)**							
*Positive*	19 (27.9)	0 (0.0)	1.00	7 (11.7)	3 (37.5)	0.08	29 (21.0)
*Negative*	49 (72.1)	2 (100)		53 (88.3)	5 (62.5)		109 (79.0)

Overall, 153 of 2,390 patients with AHD had urine LF-LAM positive test results; 6.4% (CI: 5.4–7.5). Among patients with CD4 ≤100 cells/mm^3^, 80 of 580 (13.8%; CI: 10.9–17.2), and among patients with CD4 >100 cells/mm^3^, 73 of 1,810 (4%; CI: 3.2–5.1) had positive LF-LAM test results (Table 1).

Among 100 PLHIV having CD4 ≤100 cells/mm^3^ with symptoms, the positivity was 43% (95% CI: 31.1–57.9); among 480 asymptomatic patients, the positivity was 7.7% (95% CI: 5.45–10.6). Among 101 PLHIV having CD4 >100 cells/mm^3^ with symptoms, the positivity was 26.7%; 27/101 (95% CI: 17.6–38.9); among 1,709 asymptomatic cases the positivity was 2.7%; 46/1,709 (95% CI: 2.0–3.6). Symptomatic patients in both CD4 groups had higher urine LF-LAM positivity compared to asymptomatic patients (p<0.01) (Table 2).

As per national guidelines, symptomatic PLHIV with cough underwent sputum Xpert MTB/RIF. Of the 138 PLHIV tested for sputum Xpert MTB/RIF, the overall test positivity was 21% (29/138). Among PLHIV having CD4 ≤100 cells/mm^3^, the positivity was 27.9% in symptomatic patients. Among PLHIV having CD4 >100 cells/mm^3^, the positivity was 11.7% in symptomatic patients (Table 2).

**Fig 1:**
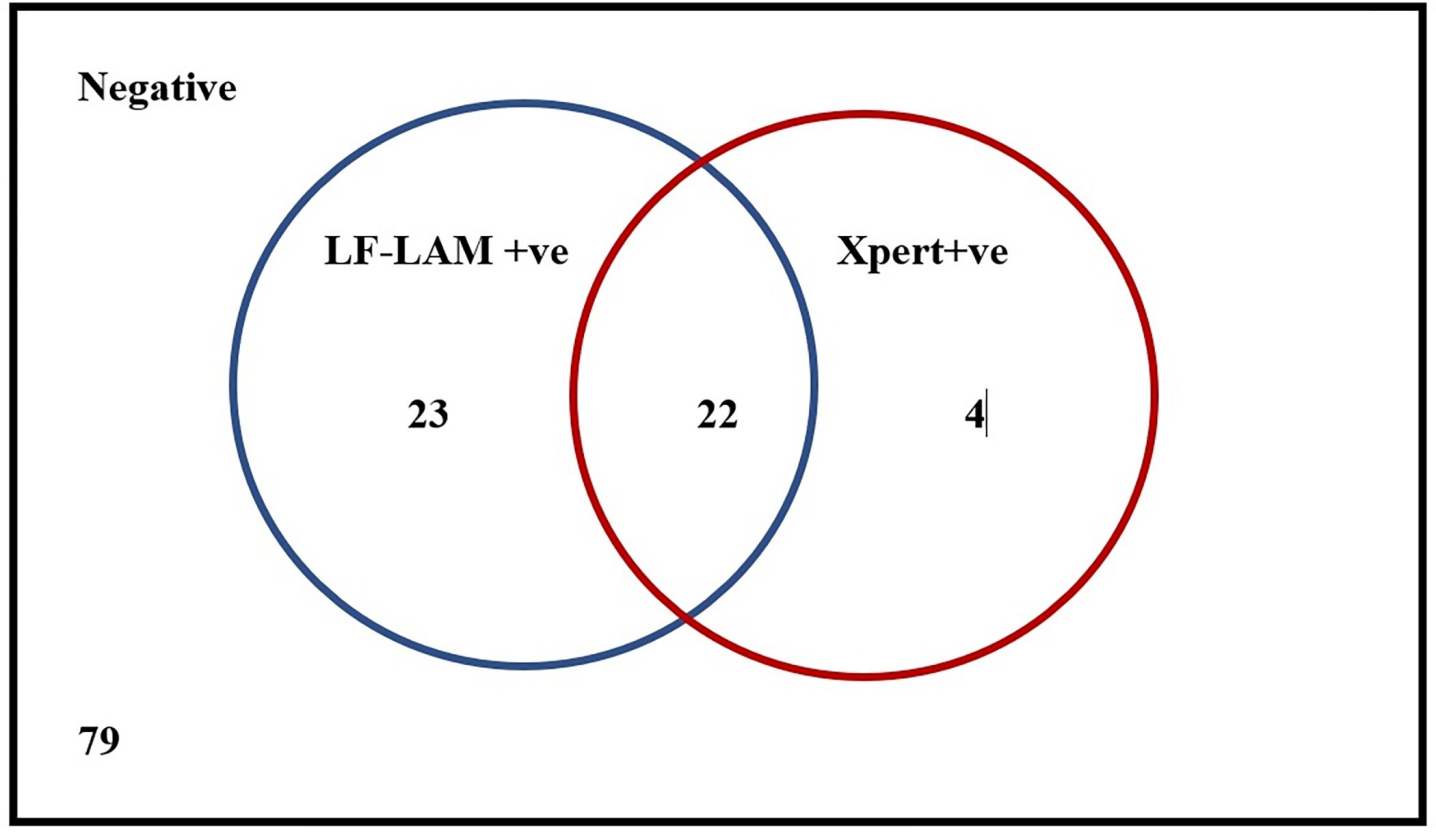
Venn diagram depicting positivity by urine LF-LAM and sputum Xpert among 128 symptomatic patients.

Of the 201 symptomatic PLHIV, 128 underwent sputum Xpert MTB/RIF test. Twleve patients with cough could not produce sputum sample and 61 patients did not have cough. Thus, of the 128 symptomatic patients with both urine LF-LAM and Xpert MTB/RIF tests, 22 patients were urine LF-LAM and Xpert MTB/RIF positive and 23 patients were additionally tested positive by urine LF-LAM test (Fig 1).

**Fig 2:**
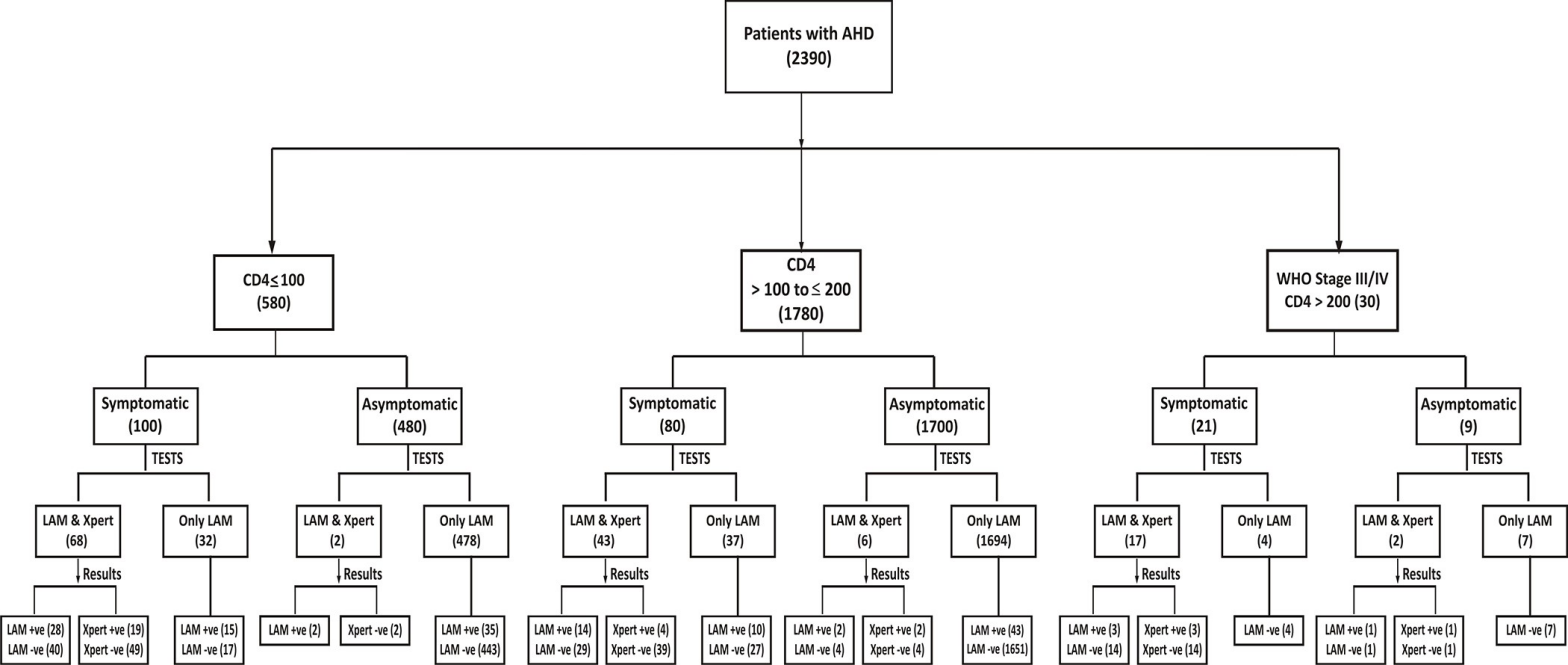
Flow diagram depicting the positivity of urine LF-LAM and sputum Xpert among 2,390 patients by CD4 count, WHO staging, and TB symptoms.

Symptomatic patients who either did not have a cough or could not expectorate were not tested by sputum Xpert MTB/RIF; 34.2% (25/73) of these patients had urine LF-LAM positive results. Among 2,189 asymptomatic patients, urine LF-LAM was done in all patients and sputum Xpert MTB/RIF was done in 10 patients. 83 patients were urine LF-LAM positive and 3 patients were sputum Xpert MTB/RIF positive. In the present study, urine LF-LAM detected an additional 131 TB cases among patients with AHD (Fig 2).

### Association of demographic, clinical and immunological characteristics with LF- LAM positivity by CD4 count–Univariate analysis

Among all 2,390 participants, females (OR:1.5; CI:1.1–2.1, p = 0.02), inpatients (OR:11.6; CI:6.3–21.4, p<0.01), ART naïve patients (OR:3.6; CI:2.5–5.2, p<0.01), PLHIV on ART for < 6 months (OR:2.0; CI:1.2–3.2, p = 0.006), and symptomatic patients (OR: 13.6; CI: 9.4–19.5, p<0.01) had higher odds to test positive for urine LF-LAM. Similarly, PLHIV in WHO stage III (OR:10.4; CI: 5.8–18.8, p<0.01), WHO stage IV (OR: 11.7; CI: 7.3–18.8, p<0.01) and patients with CD4 ≤ 100 cells/mm^3^ (OR:3.8; CI: 2.7–5.3, p<0.01) had higher odds to test positive for urine LF-LAM.

Among those with CD4 ≤100 cells/mm^3^ (n = 580), inpatients (OR:5.2; CI:2.3–11.7, p<0.01), ART naïve patients (OR:3.9; CI:2.4–6.4, p<0.01), PLHIV on ART for < 6 months (OR:2.1; CI: 1.0–4.2, p = 0.04), and symptomatic patients (OR:9.0; CI:5.4–15.2, p<0.01) had higher odds to test positive for urine LF-LAM. Similarly, PLHIV in WHO stage III (OR:10.7; CI:4.5–25.6, P<0.01) and WHO stage IV (OR: 9.3; CI:4.7–18.6, P<0.01) had higher odds of a positive urine LF-LAM.

Among those who had CD4>100 cells/mm^3^ (n = 1,810), inpatients (OR: 17.7; CI:7.0–44.8, p<0.01), symptomatic patients (OR:13.2; CI:7.8–44.8, p<0.01), PLHIV in WHO stage III (OR:7.6; CI:3.2–18.4, p<0.01), WHO stage IV (OR:10.2; CI:5.1–20.4, p<0.01) had higher odds to be tested positive for urine LF-LAM.

Among patients on treatment for more than one year, viral load suppression did not have any significant association with urine LF-LAM positivity. In patients with CD4≤100 cells/mm^3^, the odds of LAM positivity were found to be higher among patients with VL > = 1000 (OR:2.4: CI: 0.9–6.6, p = 0.09); however, the association was not statistically significant (Table 3).

**Table 3:** Association of demographic and clinical factors with urine LF- LAM positivity–Univariate analysis.

Patient characteristics	All patients (N = 2,390)					CD4 ≤ 100 (n = 580)					CD4 >100 (n = 1,810)				
Age category (years)	n	LAM Positive (%)	OR	CI 95%	P value	n	LAM Positive (%)	OR	CI 95%	P value	n	LAM Positive (%)	OR	CI 95%	P value
*18 to 24*	120	9 (7.5)	1.00			37	4 (10.8)	1			83	5 (6.0)	1.00		
*25 to 34*	320	27 (8.4)	1.14	0.52–2.49	0.75	83	13 (15.7)	1.53	0.46–5.06	0.48	237	14 (5.9)	0.98	0.34–2.81	0.97
*35 to 44*	741	46 (6.2)	0.82	0.39–1.71	0.59	178	23 (12.9)	1.22	0.40–3.78	0.72	563	23 (4.1)	0.66	0.25–1.80	0.42
*≥45*	1209	71 (5.9)	0.77	0.37–1.58	0.48	282	40 (14.2)	1.36	0.46–4.06	0.58	927	31 (3.3)	0.54	0.20–1.43	0.21
**Gender**															
*Male*	1640	93 (5.7)	1.00			381	49 (12.9)	1.00			1259	44 (3.5)	1.00		
*Female*	736	60 (8.2)	1.48	1.05–2.07	0.02	195	31 (15.9)	1.28	0.79–2.08	0.32	541	29 (4.5)	1.56	0.97–2.53	0.07
*TG*	14	0													
**Patient Setting**															
*Outpatient*	2344	134 (5.7)	1.00			554	69 (12.5)	1.00			1790	65 (3.6)	1.00		
*Inpatient*	46	19 (41.3)	11.61	6.29–21.41	<0.01	26	11 (42.3)	5.16	2.28–11.68	<0.01	20	8 (40.0)	17.69	6.99–44.76	<0.01
**ART Status**														
*On ART*	2104	106 (5.1)	1.00			452	42 (9.3)	1.00			1650	64 (3.9)			
*ART naïve*	286	46 (16.1)	3.57	2.47–5.18	<0.01	128	38 (29.7)	3.92	2.39–6.43	<0.01	160	9 (5.6)	1.49	0.73–3.05	0.28
**Duration of ART in months (n-2104)**															
*>24*	1434	72 (4.7)	1.00			299	22 (7.4)	1.00			1295	50 (3.9)	1.00		
*13 to 24*	255	14 (5.4)	0.93	0.49–1.79	0.84	62	6 (9.7)	1.13	0.41–3.12	0.82	196	8 (4.1)	0.78	0.33–1.85	0.57
*6 to 12*	139	41 (10.8)	0.94	0.40–2.19	0.88	142	32 (22.5)	0.87	0.19–3.90	0.85	238	9 (3.8)	0.94	0.33–2.67	0.91
*< 6*	276	17 (12.4)	1.97	1.21–3.21	0.006	66	15 (22.7)	2.07	1.02–4.19	0.04	71	2 (2.8)	1.34	0.65–2.79	0.43
**4S symptoms **															
*Asymptomatic*	2189	83 (3.8)	1			480	37 (7.7)	1.00			1709	46 (2.7)	1.00		
*Symptomatic*	201	70 (34.8)	13.56	9.40–19.51	<0.01	100	43 (43.0)	9.03	5.38–5.18	<0.01	101	27 (26.7)	13.19	7.77–22.39	<0.01
**VL count (n = 1682)**													
*< 1000*	1202	42 (3.5)	1			156	6 (3.8)	1.00			1046	36 (3.4)	1.00		
*≥ 1000*	480	23 (4.8)	1.39	0.83–2.34	0.21	136	12 (8.8)	2.42	0.88–6.63	0.09	344	11 (3.2)	0.93	0.47–1.84	0.83
**WHO clinical stage (n = 2,386) **															
*Stage I*	1906	83 (4.4)	1			422	36 (8.5)	1.00			1484	47 (3.2)	1.00		
*Stage II*	325	17 (5.2)	1.21	0.71–2.07	0.48	89	11 (12.4)	1.51	0.74–3.10	0.26	236	6 (2.5)	0.80	0.34–1.89	0.61
*Stage III*	59	19 (32.2)	10.43	5.79–18.80	<0.01	24	12 (50.0)	10.72	4.49–25.59	<0.01	35	7 (20.0)	7.64	3.18–18.39	<0.01
*Stage IV*	95	33 (34.7)	11.69	7.26–18.82	<0.01	43	20 (46.5)	9.32	4.68–18.58	<0.01	52	13 (25.0)	10.19	5.10–20.35	<0.01
CD4 Count (cells/mm^3)^															
*>100*	1810	73 (4.0)	1.00												
*≤100*	580	80 (13.8)	3.81	2.73–5.31	<0.01										

### Predictors of urine LF- LAM positivity stratified by CD4 count

On multivariable logistic regression, inpatients, ART naïve, symptomatic patients, patients in WHO clinical stage III & IV and patients with CD4 ≤100 cells/mm^3^ were independently associated with increased risk to test positive for urine LF-LAM (Table 4).

**Table 4:** Predictors of urine LF- LAM positivity stratified by CD4 count.

Patient characteristics	All patients (N = 2,390)				CD4 Count ≤100 (n = 580)				CD4 Count >100 (n = 1,810)		
Age category (years)	aOR	CI 95%	P value	aOR		CI 95%	P value	aOR		CI 95%	P value
*18 to 24*	1.00			1.00				1.00			
*25 to 34*	1.22	0.51–2.93	0.66	1.46		0.37–5.76	0.59	0.95		0.31–2.89	0.93
*35 to 44*	0.94	0.41–2.16	0.89	1.54		0.41–5.74	0.52	0.58		0.21–1.67	0.31
*≥45*	1.10	0.49–2.45	0.82	2.17		0.62–7.65	0.23	0.56		0.20–1.55	0.26
**Gender**											
*Male*	1.00			1.00				1.00			
*Female*	1.39	0.96–2.03	0.08	1.31		0.74–2.32	0.35	1.38		0.82–2.32	0.23
**Type of patients**											
*Outpatients*	1.00			1.00				1.00			
*Inpatients*	2.63	1.22–5.65	0.01	2.08		0.73–5.96	0.17	4.41		1.43–13.62	0.01
**Duration on ART (months)**											
*>24*	1.00			1.00							
*13–24*	0.78	0.39–1.58	0.49	0.90		0.30–2.75	0.86	0.75		0.30–1.86	0.54
*6–12*	0.86	0.36–2.09	0.74	1.11		0.23–5.27	0.89	0.86		0.29–2.55	0.78
*<6*	1.03	0.59–1.81	0.91	1.44		0.64–3.22	0.38	0.81		0.35–1.88	0.63
*ART Naïve*	1.63	1.01–2.61	0.04	3.02		1.57–5.84	<0.01	0.60		0.25–1.44	0.25
**4S Symptoms**	** **	** **									
*Asymptomatic*	1.00			1.00				1.00			
*Symptomatic*	5.58	3.45–9.03	<0.01	4.70		2.42–9.10	<0.01	7.85		3.76–16.40	<0.01
**WHO clinical stage**											
*Stage I*	1.00			1.00				1.00			
*Stage II*	0.89	0.50–1.58	0.69	0.95		0.43–2.11	0.90	0.83		0.34–1.98	0.67
*Stage III*	3.90	1.96–7.75	<0.01	5.72		2.14–15.26	<0.01	2.35		0.79–6.96	0.12
*Stage IV*	2.00	1.04–3.83	0.04	2.42		0.97–6.04	0.06	1.78		0.67–4.69	0.25
**CD4 Count (cells/mm^3^)**											
>100	1.00										
≤100	2.46	1.68–3.58	<0.01								

Among those who had CD4 ≤100 cells/mm^3^, the odds of testing positive for urine LF- LAM was 3 times (aOR: 3.0; CI:1.6–5.8, p< 0.01) higher among ART naïve, 4.7 times higher among symptomatic patients (aOR: 4.7; CI:2.4–9.1), and 5.7 times higher among PLHIV in WHO stage III (aOR: 5.7; CI: 2.1–15.3, p<0.01).

Among those who had CD4 >100 cells/mm^3^, inpatients (aOR: 4.4; CI:1.4–13.6, p = 0.01) and symptomatic patients (aOR:7.85; CI:3.76–16.4, p<0.01) had higher odds of testing positive for urine LF-LAM compared to outpatients and asymptomatic patients.

The regression analysis of only outpatients did not change the association though showed a slight increase in the strength of the association (S1 Table).

### Interview of health care providers—Benefits and challenges of using urine LF-LAM in the programme setting

The majority of ART centre staff responded that they did not face any challenges in referring patients with AHD for urine LF-LAM test on the same day. All the nurses and lab technicians were able to complete the documentation of patient referral and urine LF-LAM specimen processing on the same day. The majority of medical officers were satisfied with the availability of urine LF-LAM tests to assist in the diagnosis of TB among clinically ill patients. Some of the responses from the ART centre staff are presented below:

“*We used to face challenges in the diagnosis of TB with clinical symptoms when tests like Xpert or X-Ray chest were negative; the positive LAM test provided clear direction for a clinician to decide the line of management in such cases*”

- Medical Officer at an ART Centre

“*Few cases who were non-responders to other line of treatment but were screened for urine LF-LAM test as a final ray of hope*, *have shown wonders in clinical conditions after starting anti-tubercular treatment*”.

- Medical Officer at an ART Centre

“*Urine LF-LAM test has definitely provided an additional yield among TB asymptomatic patients*”.

- Nurse at an ART Centre

“*We experienced difficulties in convincing TB symptomatic patients for anti TB treatment in the absence of microbiological or other evidence*. *Now with urine LF-LAM*, *we could show them the test result and easily convince them for anti TB treatment*”

- Nurse at an ART Centre

*“It is a very good test from patient’s point of view*, *the testing process is simple and easy, results are available very quickly, there are no issues with reporting”*

-Lab Technician at an ART Centre

ART centres staff shared some operational challenges in the referral of asymptomatic patients for urine LF-LAM test and in collection and disposal of urine samples at some high caseload centres.

## Discussion

This key study from India explored the utility and acceptability of urine LF-LAM test among patients with AHD at public ART centres. In the current study, the overall urine LF-LAM positivity was 6.4%; this proportion was higher (13.8%; 80/580) in immuno-compromised patients with CD4 ≤100 cells/mm^3^ as compared to 4% (73/1,810) in patients with CD4 >100 cells/mm^3^.

Among PLHIV with CD4 ≤100 cells/mm^3^, the urine LF-LAM positivity indicated a significant difference between symptomatic (43%) and asymptomatic patients (7.7%). The results are in agreement with a study conducted in Malawi and Mozambique that indicated a positivity of 40.8% among symptomatic patients with CD4 ≤100 cells/mm^3^ [22]. A study by Huerga et al (2020) in Malawi showed a positivity of 31.6% among symptomatic patients with CD4 ≤100 cells/mm^3^ [23]. A prospective, observational study conducted in Mozambique by Huerga et al (2020) showed a positivity of 11.9% among PLHIV with CD4 <100 cells/mm^3^ with a significant difference between symptomatic (18.5%) and asymptomatic patients (6.6%) [24].

Among PLHIV with CD4 >100 cells/mm^3^, the current study found a urine LF-LAM positivity of 26.7% in symptomatic patients and 2.7% in asymptomatic patients. The findings corroborate with a study conducted in Malawi by Huerga et al (2019) that showed a positivity of 28.1% in symptomatic PLHIV with CD4 100–199 cells/mm^3^ [22]. Another study in Malawi by Huerga et al (2020) reported a positivity of 16.2% among symptomatic patients with CD4 100–199 cells/mm^3^ [23].

The sputum Xpert MTB/RIF positivity among symptomatic patients in the current study was 20.3% (26/128); it was 27.9% among symptomatic patients with CD4≤100 cells/mm^3^ and 11.7% in patients with CD4>100 cells/mm^3^. In India, sputum Xpert MTB/RIF is the standard molecular diagnostic test advocated for TB diagnosis in symptomatic PLHIV [15]. Among the symptomatic patients who underwent both the tests in the present study, the urine LF-LAM was found to provide an incremental yield of 23 TB cases in accordance with other studies [25, 26]. This is an important finding for HIV-TB control programs in the country as these patients might have otherwise missed their TB diagnosis in the routine program setting.

Nearly 36% (73/201) of the symptomatic AHD patients had no cough or could not produce sputum for testing (Fig 2). The urine LF-LAM test positivity among these symptomatic patients (25 cases) signifies the important role of this test in the diagnosis of pulmonary/extrapulmonary TB among AHD patients. Existing literature highlighted that the diagnostic yield of urine LF-LAM is unrelated to respiratory symptoms as LAM positivity may be a marker of greater disease dissemination and severity [26]. These findings underscore the importance of urine LF-LAM test in the diagnosis of extrapulmonary TB in symptomatic PLHIV considering the operational challenges or inaccessibility of other diagnostic modalities (e.g., CT scan) in most of the primary and secondary health care settings of the country. The addition of urine LF-LAM test in the current diagnostic algorithm can aid in rapid diagnosis of TB among symptomatic PLHIV with advanced immunosuppression at peripheral health settings.

In the present study, the urine LF-LAM positivity of 7.7% (37/480) was found among asymptomatic patients with CD4 ≤100 cells/mm^3^. WHO recommends urine LF-LAM test among patients with AHD with CD4 ≤100 cells/mm^3^ irrespective of signs/symptoms of TB and the study finding of 7.7% positivity in this group confirms the programmatic importance of urine LF-LAM in rapid diagnosis of TB. Though WHO recommends against using urine LF-LAM in the diagnosis of TB among patients with CD4 count 100–200 cells/mm^3^ without TB symptoms in outpatient settings due to low sensitivity, the present study found an incremental positive yield of 45 TB cases (2.6%; 45/1,700) in this group. Existing literature indicates a lower sensitivity for WHO four-symptom screening for TB disease among PLHIV on ART and therefore, urine LF-LAM test may help in systematic screening of TB among patients with advanced immune suppression, [6, 27, 28]. Prospective cohort studies among patients with CD4 100–200 cells/mm^3^ would be beneficial for additional evidence.

As per the available literature on urine LF-LAM, several hypotheses may explain the higher sensitivity of urine LAM detection in PLHIV with AHD, including higher bacillary burden and antigen load, greater likelihood of genitourinary tract TB involvement and greater glomerular permeability that leads to increased antigen levels in urine [29, 30]. This could explain the additional yield of TB cases in the present cohort of patients with AHD and highlights the role of testing with urine LF-LAM to assist in the early diagnosis of TB among patients with AHD in India, a high TB burden country.

In the current study, urine LF-LAM positivity among ART naïve PLHIV was 16.1% compared to those on treatment (5.1%) and the significant difference persisted among symptomatic (44.8% vs 29.9%) and asymptomatic cases (7.3% vs 3.4%). Few other studies have highlighted the usefulness of urine LF-LAM for TB diagnosis in ART naïve PLHIV with AHD [31–34]. The higher positivity among ART naïve PLHIV in the present study underscores the necessity of urine LF-LAM assay as a rule-in-test for screening among all newly diagnosed patients with AHD at the time of ART initiation.

PLHIV on ART for > 12 months showed a protective effect with lower urine LF-LAM positivity, corroborating with existing literature [35–37]. However, ensuring documentation of weight and TB symptoms during each visit to ART centre for intensified TB screening for all PLHIV on ART and inclusion of urine LF-LAM test in combination with the standard molecular diagnostic tests in the current diagnostic algorithm among AHD patients may help in early diagnosis of TB.

We found a higher urine LF-LAM positivity in inpatients, ART naïve, those who are in ART for a shorter duration, symptomatic patients, and among those who are in WHO clinical stage III and IV of HIV disease in agreement with the existing literature. [24, 26, 38]. This provides further evidence for using urine LF-LAM tests among the AHD patients in the current programmatic settings.

The user perspectives on the acceptability of urine LF-LAM test among the ART centre staff highlighted the ease of use, availability of quick results and the unmet need for a diagnostic test in the difficult to diagnose presumptive TB patients with AHD. The wider availability of the point of care urine test for processing convenient patient samples through appropriate training of health care staff at peripheral health care settings can definitely aid in rapid TB diagnosis among patients with advanced HIV disease.

### Limitations

This study was cross-sectional and provides information on urine LF-LAM tests performed only once during the period. Repeat urine LF-LAM test may not have been performed for the enrolled asymptomatic negative patients subsequently becoming symptomatic during the study period. Additional confirmatory tests for urine LF-LAM positive patients, beyond the standard molecular diagnostic tests, were not performed as per the existing WHO and National guidelines for TB diagnosis among PLHIV. The perspectives of patients or caregivers about the urine LF-LAM test for TB diagnosis were not sought. The impact analysis of TB treatment among the diagnosed TB patients is ongoing.

## Conclusion

The study demonstrates the utility of urine LF-LAM test in the diagnosis of HIV associated TB in patients with AHD. The simple, user-friendly, and point of care test was acceptable as part of the routine care at public ART centres in Mumbai, India.

Urine LF-LAM test contributed to an incremental yield in TB diagnosis in combination with the standard molecular diagnostic tests among symptomatic and asymptomatic patients with AHD. Urine LF-LAM test may be offered for all treatment naive patients with AHD at ART centres during baseline screening. Inclusion of urine LF-LAM test in combination with the standard molecular diagnostic tests in the current diagnostic algorithm may facilitate rapid TB diagnosis among AHD patients.

## Supporting information

S1 Table Factors associated with LAM positivity among PLHIV with advanced HIV disease (AHD)–Only outpatient cases (N = 2,344). (DOCX)
