# Supplementary material for: Utility of the lateral flow urine lipoarabinomannan tuberculosis assay in patients with advanced HIV disease at antiretroviral therapy centres in Mumbai, India
Source: PLoS One. 2022 Sep 14;17(9):e0273970. doi: 10.1371/journal.pone.0273970 (PMC9473623; doi:10.1371/journal.pone.0273970)
Supplement: S1 Table — (DOCX) [file pone.0273970.s001.docx]

**SUPPORTING FILES - APPENDIX**

**Table 5 - Factors associated with LAM positivity among PLHIV with and advanced**

**HIV disease (AHD) – Only OP cases (N = 2,344)**

| **Description** | **All Cases**  **(N =2,344)** | | | **CD4 Count <=100 (n =554)** | | | **CD4 Count >100 (n =1,790)** | | |
| --- | --- | --- | --- | --- | --- | --- | --- | --- | --- |
| **Age category in years** | aOR | CI 95% | P  value | aOR | CI 95% | P  value | aOR | CI 95% | P  value |
| ***Up to 24*** | 1.00 |  |  | 1.00 |  |  | 1.00 |  |  |
| ***25 to 34*** | 1.49 | 0.58 – 3.77 | 0.41 | 1.54 | 0.37 – 6.37 | 0.55 | 1.22 | 0.36 – 4.18 | 0.75 |
| ***35 to 44*** | 1.06 | 0.44 – 2.58 | 0.89 | 1.52 | 0.39 – 5.87 | 0.55 | 0.68 | 0.21 – 2.19 | 0.52 |
| ***>=45*** | 1.40 | 0.60 – 3.30 | 0.44 | 2.36 | 0.66 – 8.50 | 0.19 | 0.78 | 0.25 – 2.44 | 0.67 |
| **Gender** |  |  |  |  |  |  |  |  |  |
| ***Male*** | 1.00 |  |  | 1.00 |  |  | 1.00 |  |  |
| ***Female*** | 1.50 | 1.01 – 2.22 | 0.04 | 1.54 | 0.85 – 2.79 | 0.15 | 1.44 | 0.84 – 2.47 | 0.18 |
| ***TG*** | - |  |  | - |  |  | - |  |  |
| **Duration of ART in months** | | |  |  |  |  |  |  |  |
| **>24** | 1.00 |  |  | 1.00 |  |  | 1.00 |  |  |
| **13 – 24** | 0.80 | 0.39 – 1.67 | 0.56 | 1.10 | 0.35 –3.43 | 0.87 | 0.70 | 0.27 – 1.86 | 0.48 |
| **6 - 12** | 0.94 | 0.38 –2.30 | 0.89 | 1.37 | 0.28 – 6.70 | 0.70 | 0.83 | 0.28 –2.52 | 0.75 |
| **<6** | 1.14 | 0.63 – 2.07 | 0.66 | 1.50 | 0.62 – 3.64 | 0.37 | 1.04 | 0.45 –2.39 | 0.92 |
| **ART Naïve** | 1.67 | 1.01 – 2.75 | 0.04 | 3.34 | 1.65 – 6.74 | <0.01 | 0.60 | 0.23 – 1.54 | 0.29 |
| **4S Symptoms** |  |  |  |  |  |  |  |  |  |
| ***Asymptomatic*** | 1.00 |  |  | 1.00 |  |  | 1.00 |  |  |
| ***Symptomatic*** | 6.47 | 3.96 –  10.57 | <0.01 | 5.14 | 2.59 –  10.16 | <0.01 | 9.52 | 4.58 –  19.77 | <0.01 |
| **WHO clinical stage of HIV disease** | | |  | - | - | - | - | - | - |
| ***Stage I*** | 1.00 |  |  | 1.00 |  |  | 1.00 |  |  |
| ***Stage II*** | 0.89 | 0.49 – 1.60 | 0.69 | 0.95 | 0.42 – 2.12 | 0.90 | 0.85 | 0.35 – 2.05 | 0.72 |
| ***Stage III*** | 4.36 | 2.17 – 8.77 | <0.01 | 5.93 | 2.19 –  16.07 | <0.01 | 3.02 | 1.02 – 8.91 | 0.05 |
| ***Stage IV*** | 2.00 | 0.99 – 4.03 | 0.05 | 2.61 | 0.94 – 7.21 | 0.07 | 1.54 | 0.55 – 4.35 | 0.41 |
| **CD4 Count** | | |  |  |  |  |  |  |  |
| ***>100*** | 1.00 |  |  |  |  |  |  |  |  |
| ***<=100*** | 2.48 | 1.67 – 3.71 | 0.00 |  |  |  |  |  |  |
